# Draft Genome Sequence of *Citrobacter rodentium* DBS100 (ATCC 51459), a Primary Model of Enterohemorrhagic *Escherichia coli* Virulence

**DOI:** 10.1128/genomeA.00415-15

**Published:** 2015-05-07

**Authors:** Abigail Lenz, Jeffrey Tomkins, Andrew J. Fabich

**Affiliations:** Department of Biology and Chemistry, Liberty University, Lynchburg, Virginia, USA

## Abstract

*Citrobacter rodentium* is a Gram-negative bacterium which causes transmissible murine colonic hyperplasia and models the virulence of enterohemorrhagic *Escherichia coli in vivo*. Thus, *C. rodentium* is used to study human gastrointestinal disease. We present the draft genome sequence of *C. rodentium* strain ATCC 51459, also known as DBS100.

## GENOME ANNOUNCEMENT

*Citrobacter rodentium* is a Gram-negative bacterium which causes transmissible colonic hyperplasia in mice. Mice are the preferred model for investigating human gastrointestinal disease because mice and humans have similar gastrointestinal tracts. The *C*. *rodentium* mouse model is well established for research to understand enterohemorrhagic *Escherichia coli* (EHEC) virulence factors since there is no good model to study EHEC virulence factors *in vivo* (1). EHEC is the leading cause of diarrhea in humans, but it is not pathogenic to mice, thus making the use of *C. rodentium* essential for understanding EHEC virulence. Both EHEC and *C. rodentium* use the same pathogenicity island, called the locus of enterocyte effacement, which causes the diagnostic attaching and effacing lesion (2, 3). The most widely studied strains of *C. rodentium* are ICC168 and DBS100 (ATCC 51459), which were both isolated from an outbreak of diarrhea in Swiss-Webster mice at Yale University School of Medicine in 1972 (4–6). Though the entire genome of ICC168 has been sequenced (3), only small portions of the DBS100 have been previously sequenced. Knowing that genomic diversity exists among *C. rodentium* isolates over a relatively short period of time, we decided to sequence the *C. rodentium* strain DBS100 deposited as ATCC 51459. We introduce the first draft genome of DBS100 under the designation ATCC 51459.

Genomic DNA was purified from *C. rodentium* strain DBS100 with the Qiagen DNeasy kit no. 69504. The genome was sequenced using the Illumina MiSeq sequencing system, which produced 2.2-M random paired-end reads of 32 to 300 bases per read. After quality analysis with FastQC, reads were assembled using Spades (v 3.0), Velvet (v 1.1.06), and Bio Star, with standard parameters for MiSeq data obtained from *E. coli*-like genomes. The highest quality genome assembly utilized for public database submission was produced using Bio Star and contains 361 total contigs of 411 to 138,720 bp in length. The mean contig length is 14,919 bp, median contig length is 5,883 bp, and total assembly size is 5,385,810 bp (contig *N*_50_, 34,885).

The submitted genome assembly was annotated using the NCBI Prokaryotic Genomes Annotation Pipeline (2.9, rev. 456657). The annotation method utilized a best-placed reference protein set (GeneMarkS+). Identified features include 5,268 genes, 4,828 CDS, 305 pseudogenes, 1 clustered regularly interspaced short palindromic repeat (CRISPR) array, 38 rRNAs (5S, 16S, and 23S), 80 tRNAs, 17 noncoding RNAs (ncRNAs), and 103 frame-shifted genes.

### Nucleotide sequence accession numbers. 

This whole-genome shotgun project using the strain name ATCC 51459 has been deposited at DDBJ/EMBL/GenBank under the accession number JXUN00000000. The version described in this paper is JXUN01000000. Sequence data are also linked under BioProject PRJNA261533 and BioSample SAMN03115005. Raw MiSeq fastq reads were deposited in the Sequence Read Archive (SRA) at http://www.ncbi.nlm.nih.gov/sra.
